# Development of an artificial intelligence-based method for the diagnosis of the severity of myxomatous mitral valve disease from canine chest radiographs

**DOI:** 10.3389/fvets.2023.1227009

**Published:** 2023-09-22

**Authors:** Carlotta Valente, Marek Wodzinski, Carlo Guglielmini, Helen Poser, David Chiavegato, Alessandro Zotti, Roberto Venturini, Tommaso Banzato

**Affiliations:** ^1^Department of Animal Medicine, Production and Health, University of Padua, Padua, Italy; ^2^Department of Measurement and Electronics, AGH University of Science and Technology, Krakow, Poland; ^3^Information Systems Institute, University of Applied Sciences—Western Switzerland (HES-SO Valais), Sierre, Switzerland; ^4^AniCura Clinica Veterinaria Arcella, Padua, Italy

**Keywords:** dog, heart, artificial intelligence, myxomatous mitral valve disease, radiology

## Abstract

An algorithm based on artificial intelligence (AI) was developed and tested to classify different stages of myxomatous mitral valve disease (MMVD) from canine thoracic radiographs. The radiographs were selected from the medical databases of two different institutions, considering dogs over 6 years of age that had undergone chest X-ray and echocardiographic examination. Only radiographs clearly showing the cardiac silhouette were considered. The convolutional neural network (CNN) was trained on both the right and left lateral and/or ventro-dorsal or dorso-ventral views. Each dog was classified according to the American College of Veterinary Internal Medicine (ACVIM) guidelines as stage B1, B2 or C + D. ResNet18 CNN was used as a classification network, and the results were evaluated using confusion matrices, receiver operating characteristic curves, and t-SNE and UMAP projections. The area under the curve (AUC) showed good heart-CNN performance in determining the MMVD stage from the lateral views with an AUC of 0.87, 0.77, and 0.88 for stages B1, B2, and C + D, respectively. The high accuracy of the algorithm in predicting the MMVD stage suggests that it could stand as a useful support tool in the interpretation of canine thoracic radiographs.

## 1. Introduction

Myxomatous mitral valve disease (MMVD) is the most common acquired cardiac disease in small to medium-sized adult dogs. Myxomatous mitral valve disease is potentially a serious threat to canine health because in its final stages it can lead to congestive heart failure (CHF). According to the *American College of Veterinary Internal Medicine* (ACVIM), MMVD can be classified as: preclinical (referred to as stages B1 and B2) when structural cardiac abnormalities associated with MMVD, but no clinical signs of heart failure (HF), are evident or decompensated (stages C and D) when current or past signs of HF are reported (1). Early identification of MMVD can help both to delay the onset and to efficiently manage decompensated HF (2).

Chest radiographs should always be performed as part of the routine clinical evaluation of patients with suspected cardiac disease to detect concurrent lung disease (e.g., interstitial lung disease, bronchial disease, etc). Furthermore, the correct evaluation of the cardiac silhouette on thoracic radiographs, in particular the screening for left atrial and left ventricular enlargement, carinal elevation, presence of an alveolar and/or venous pulmonary pattern, can aid in staging the severity of MMVD (3). However, such an interpretation can be challenging and requires the presence of an experienced radiologist and/or cardiologist in the clinic. Furthermore, echocardiography is the gold-standard imaging technique for the detection and staging of heart disease. Unfortunately, it is time-consuming and requires trained operators and specific equipment. For the above reasons, it is not always easy to perform in general practice.

In recent years, several tools based on artificial intelligence (AI) have been released to support clinicians in their day-to-day medical routine, both for human and veterinary medicine. Along with this expanding development, the use of AI-based tools for cardiovascular imaging has also increased (4, 5). In humans, these techniques were developed to predict heart disease, to improve image interpretation, and, lastly to increase the quality of patient care (6). Recently, a deep learning (DL)-based model for the diagnosis of mitral regurgitation from thoracic radiographs has been developed in humans (7).

In veterinary medicine, AI-based algorithms has been developed for the detection of some radiographic findings, such as pneumothorax, pulmonary masses or pleural effusion (8–10). Furthermore, some articles proposing tools for the automatic evaluation of cardiac silhouette from canine thoracic radiographs have been published. Detection of cardiomegaly has been investigated in both dogs and cats (9, 11–13). In addition, AI-based tools have been proposed in assessing cardiogenic pulmonary edema secondary to MMVD and in identifying left atrium (LA) dilation on canine thoracic radiographs (14, 15). However, radiographs in the above studies were classified according to: the radiographic appearance of the cardiac silhouette (9); or the echocardiographic evaluation of LA dilation (13, 14). The ACVIM guidelines for patient classification were never used in the above studies. Therefore, the aim of this study was to develop and test a convolutional neural network (CNN) for classifying, from canine thoracic radiographs, the different stages of MMVD as set out in the ACVIM guidelines.

## 2. Materials and methods

### 2.1. Study design and database creation

In this retrospective multicentric study, data were collected from the Veterinary Teaching Hospital of the University of Padua and from the AniCura Arcella Veterinary Clinic (Padua). Dogs over 6 years of age with concomitant thoracic radiographs and echocardiographic examination were extracted from the medical database of the two centres. Cases admitted between July 2012 and December 2022 were included. The animals were classified to the ACVIM guidelines (2019) (1), as described below: asymptomatic dogs with no signs of radiographic or echocardiographic cardiac remodelling (e.g., left atrial and ventricular enlargement) were classified as B1; dogs were classified as B2 if cardiomegaly with left atrial and ventricular enlargement was evident; animals with at least one episode of pulmonary edema and/or pleural effusion due to CHF were considered stage C; symptomatic dogs refractory to standard cardiac treatment were classed as stage D. Lastly, if no abnormalities were found in clinical examination, echocardiographies or thoracic radiographs, the dogs were classified as healthy. Dogs affected by other heart or systemic diseases were excluded.

### 2.2. Radiographic and echocardiographic findings

Right or left lateral (RL or LL) and/or a dorso-ventral (DV) or ventro-dorsal (VD) projections were obtained for each dog. All radiographs in which the cardiac silhouette was not perfectly visible due to a severe abnormal pulmonary pattern were discarded, and only radiographs with a clearly defined cardiac silhouette was clearly defined were included in the final database.

Experienced operators (CG, HP, and DC) performed transthoracic echocardiographic examination in the right and left parasternal windows using standard views (16) and by means of commercially available ultrasound scanners (CX50, Philips, Eindhoven, The Netherlands; Philips Affiniti 50, Italy).

Echocardiographic assessment of cardiomegaly was based on left atrium (LA) and left ventricle (LV) dimensions. Early diastolic LA diameter and aortic root diameter (Ao) were measured from the short-axis view at the heart base level in the right parasternal window. The left atrium to aortic root ratio (LA/Ao) was calculated and LA dilation was considered when LA/Ao ≥1.6 (17).

Left ventricular internal diastolic diameter (LVIDd) was measured in the right parasternal window from the M-mode short axis view at the level of the cordae tendinae; this was then normalised for body weight (LVIDd-N). Values of LVIDd-N ≥1.7 were representative of LV dilation (18).

### 2.3. Image analysis

The radiographic images were stored in DICOM format and then converted to mha format for analysis. All images were resampled at a 224 × 224 resolution, and the intensity range was normalised to (0–1). CNN ResNet18 was used as a classification network due to the relatively small dataset size (compared to other computer vision datasets) and the lack of improvement compared to larger networks (ResNet50, EfficientNet, Vision Transformer). ResNet18 was fine-tuned from ImageNet pre-trained weights by unfreezing all layers. Fine-tuning only the last layer was suboptimal because the low-level radiographic features differ substantially from the ImageNet features.

The training was performed using five-fold cross-validation, separately, for the lateral and DV or VD projections. Each fold was trained for the same number of epochs (3000), and the state of the model from the last training epoch was utilised for further evaluation. The number of epochs was chosen experimentally until convergence. The number of epochs was relatively high due to heavy data augmentation and small epoch size. The validation set was not used to make any decisions during the training procedure. The objective function was the cross-entropy loss, the optimiser was the AdamW algorithm, and the learning rate scheduler was based on exponential decay. The training set was online augmented by random: (i) horizontal and vertical flipping, (ii) affine transformations, (iii) elastic transformations, (iv) contrast changes, (v) Gaussian blur, (vi) pixel dropout, (vii) random sharpening, using the Albumentations library. The class imbalance was addressed by oversampling the minority classes for the training cases to achieve balance in the training batches. All experiments were implemented using the PyTorch library and performed on a single NVIDIA A100 GPU. The results were evaluated using confusion matrices, receiver operating characteristic (ROC) curves, t-distributed stochastic neighbour embedding (t-SNE) and uniform manifold approximation and projection (UMAP).

## 3. Results

### 3.1. Database

The database consisted of 1,242 (793 from the Veterinary Teaching Hospital and 449 from the Arcella Veterinary Clinic) radiographs in total, including 728 (58.6%) (381 from the Veterinary Teaching Hospital and 347 from the Arcella Veterinary Clinic lateral and 514 (41.4%) (412 from the Veterinary Teaching Hospital and 102 from the Arcella Veterinary Clinic) DV or VD projections.

Due to the relatively low number of healthy control cases (84 and 53 lateral and DV/VD radiographic views, respectively), these were not included in the database for the final analysis. Similarly, since only a few radiographs were classified as stage D (27 and 15 lateral and DV/VD radiographic views, respectively) dogs classified as stage C or D were merged in the database and named as the C + D group.

The lateral and DV or VD views were analysed separately. Two hundred and thirty-three (32%) lateral radiographs were classified as B1, 165 (22.7%) as B2, and 330 (45.3%) as C + D. One hundred and seventy-nine (34.8%) DV or VD radiographs were classed as B1, 127 (24.7%) were classified as B2, and 208 (40.5%) as C + D.

### 3.2. Classification results

#### 3.2.1. ROC curve

**Figure 1:**
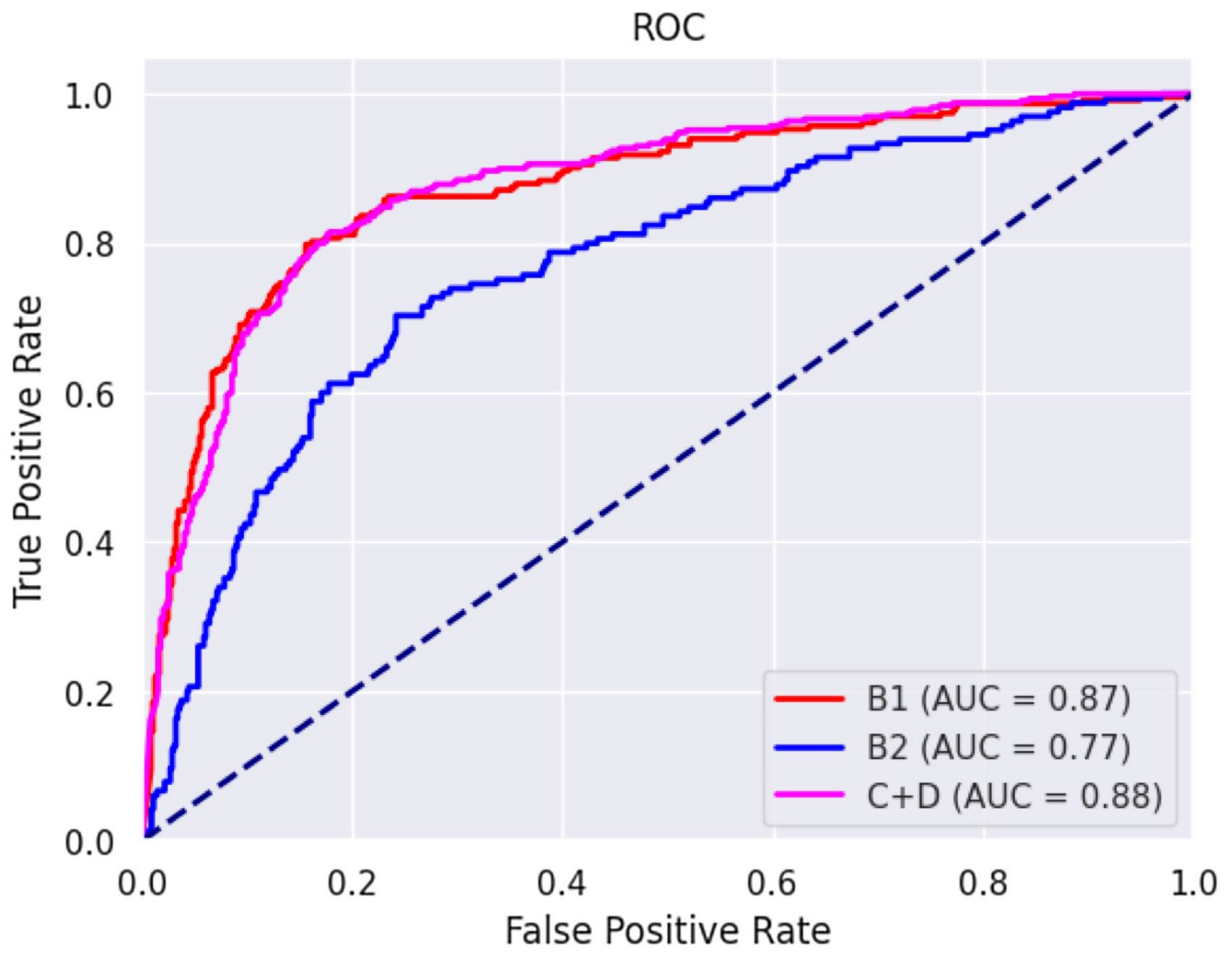
Receiver operating characteristic curve of right and left lateral radiographic views. The area under the curve was 0.87, 0.77, and 0.88 for B1, B2, and C + D, respectively.

**Figure 2:**
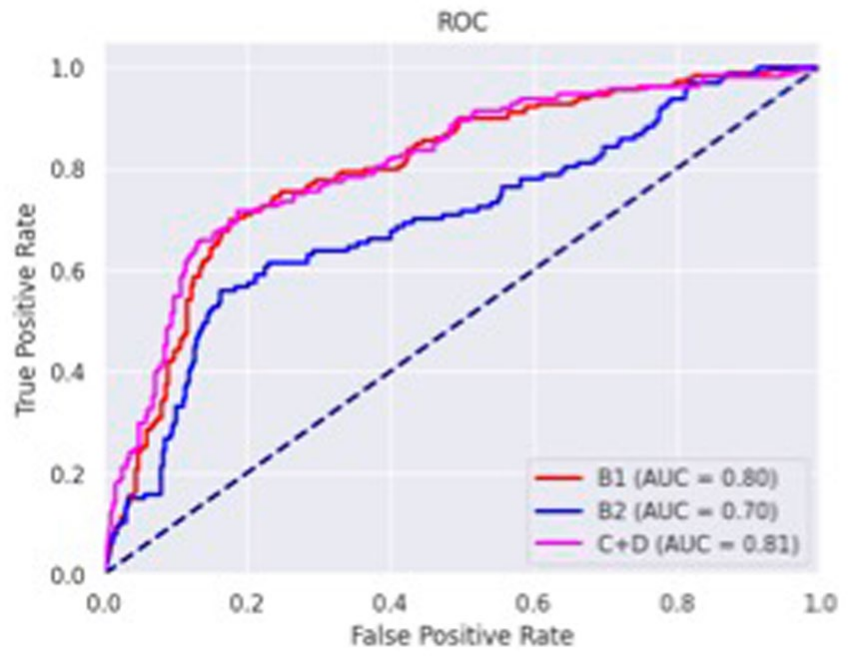
Receiver operating characteristics curve of dorso-ventral or ventro-dorsal radiographic views. The area under the curve was 0.80, 0.70 and 0.81 for B1, B2, and C + D, respectively.

The confusion matrices for classifying the lateral and DV or VD radiographs are reported in Tables 1, 2, respectively. The ROC curves are reported in Figures 1, 2 for the lateral and DV or VD views, respectively. The area under the curve (AUC) showed a good performance of the developed algorithm in determining MMVD stages from lateral radiographic views, with an AUC of 0.87, 0.77, and 0.88 for the B1, B2, and C + D groups, respectively. Instead, the AUCs for the DV or VD images were 0.80, 0.70 and 0.81 for the B1, B2, and C + D groups, respectively.

**Table 1:** Confusion matrix of right and left lateral radiographic views.

	Predicted labels			
		B1 group	B2 group	C + D group
Real labels	B1 group	167	38	28
	B2 group	39	88	38
	C + D group	22	46	262

**Table 2:** Confusion matrix of dorso-ventral or ventro-dorsal radiographic views.

	Predicted labels			
		B1 group	B2 group	C + D group
Real labels	B1 group	125	29	25
	B2 group	32	69	26
	C + D group	31	35	142

The overall precision in classifying the lateral radiographs was 71%, with a precision of 73, 51% and 80% for B1, B2, and C + D, respectively. The macro average and the weighted average were 68% and 71%, respectively.

A lower accuracy was obtained for DV or VD radiographs, with an overall accuracy of 65% and a precision of 66%, 52%, and 74% for the B1, B2, and C + D groups, respectively. The macro average and weighted average were 64% and 66%, respectively.

#### 3.2.2. Reduction in dimensionality

**Figure 3:**
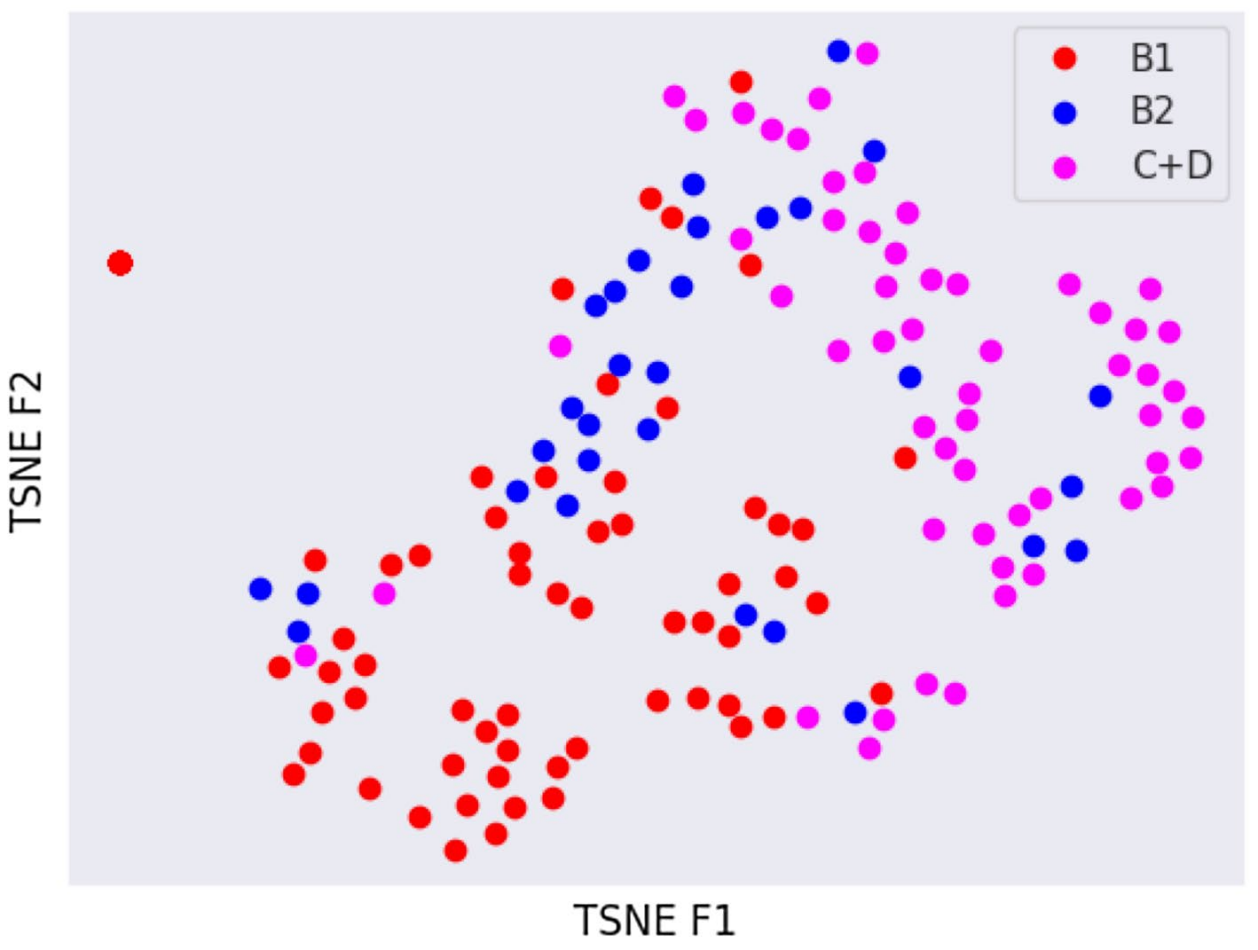
Visual distribution of latent spaces using t-SNE graph of right and left lateral radiographic views.

**Figure 4:**
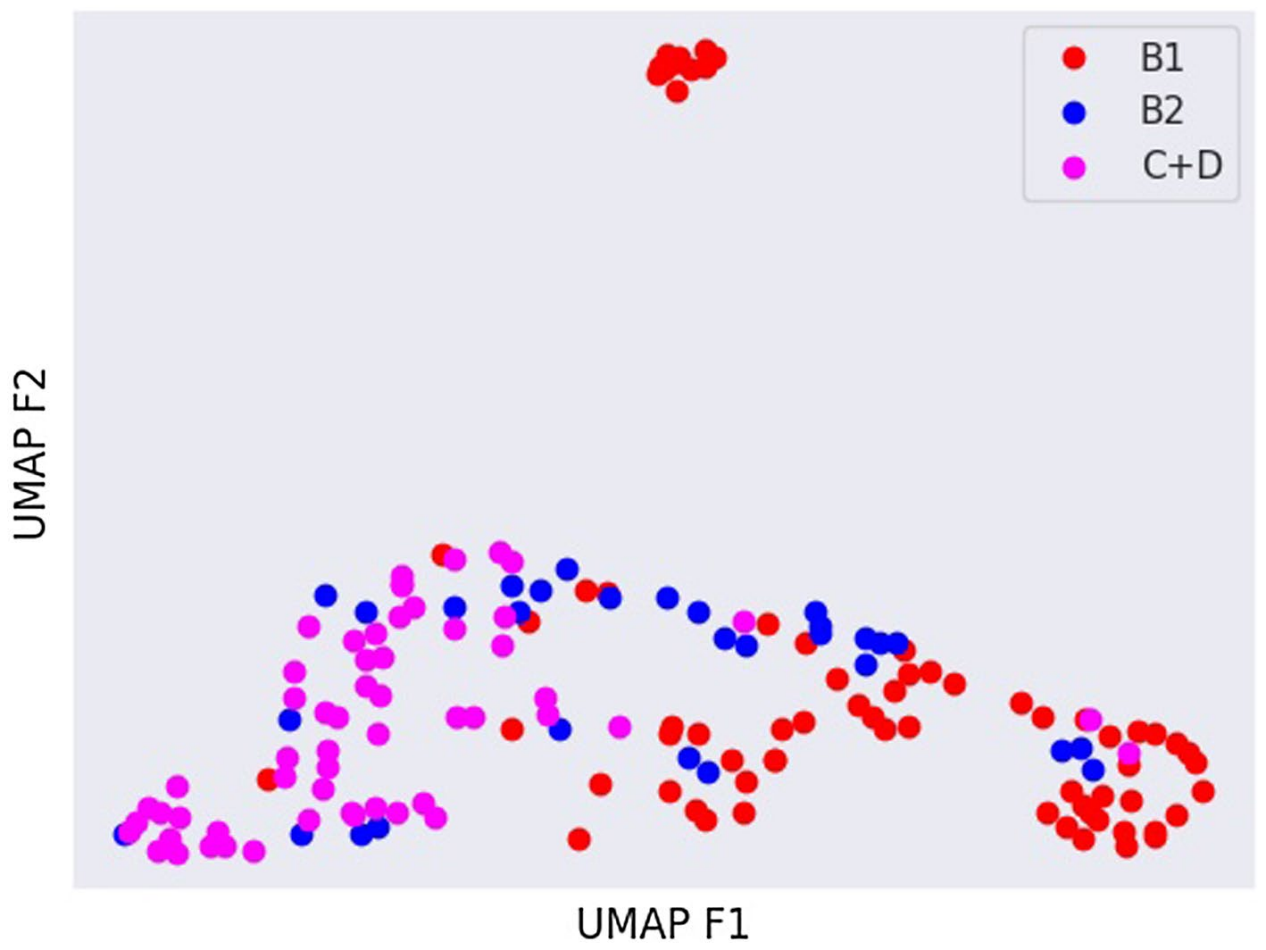
Visual distribution of latent spaces using UMAP graph of dorso-ventral or ventro-dorsal radiographic views.

**Figure 5:**
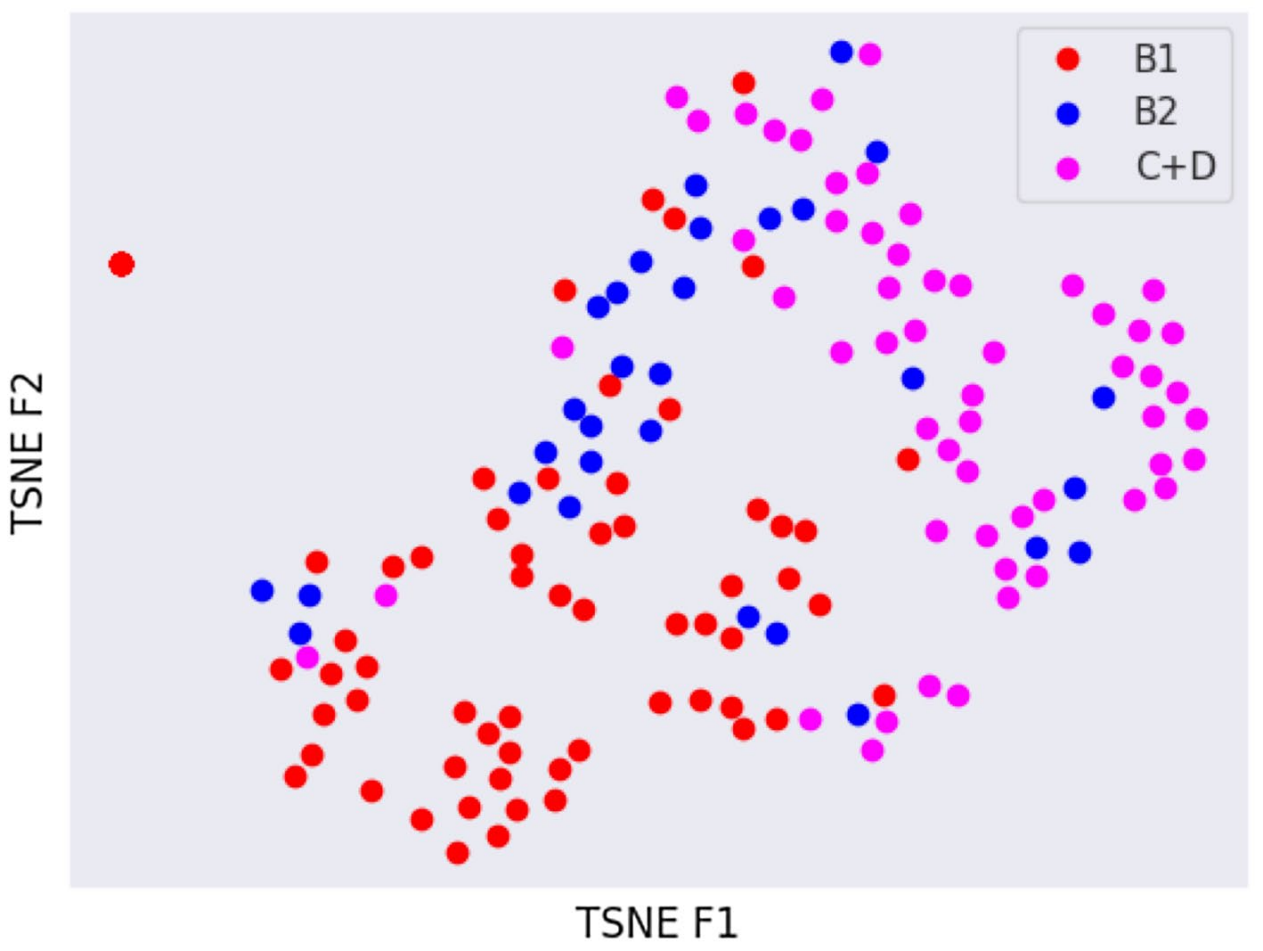
Visual distribution of latent spaces using t-SNE graph of right and left lateral radiographic views.

**Figure 6:**
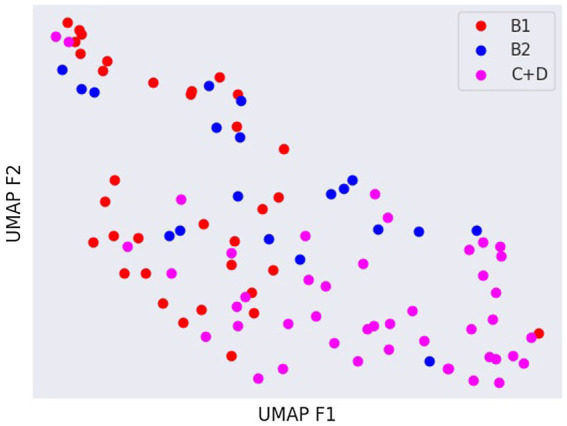
Visual distribution of latent spaces using UMAP graph of dorso-ventral or ventro-dorsal radiographic views.

Latent spaces are present after dimensionality reduction using t-SNE and UMAP in Figures 3, 4 for the right and left lateral projections, and in Figures 5, 6 for the DV or VD projections. A latent space is a set of the features describing the input images after an automatic feature extraction by the deep neural network. The dimensionality of the latent space may be further reduced by dimensionality reduction algorithms such as principal component analysis (PCA), t-SNE or UMAP, while preserving as much variance as possible. In practice, nonlinear learning-based methods such as t-SNE or UMAP are superior compared to PCA. These algorithms enable intuitive visualisation of the features distribution in low-dimensional space (e.g., 2D or 3D). Figures 3–6 illustrate that the classification features are distributed in a way that places the B2 cases between the B1 and C + D cases, even though the ground truth was not annotated using the radiography images. This confirms that the features learned by the network are indeed connected with disease severity and are not dominated by bias related to confounders caused by radiographs acqusition. Cases classed the same ACVIM group are shown close to each other. The same behaviour is achieved using both t-SNE and UMAP dimensionality reduction.

## 4. Discussion

In this study, an AI-based algorithm is proposed to predict the stage of MMVD from canine thoracic radiographs. The high accuracy of the algorithm in predicting the MMVD stage suggests that it could potentially support general practitioners in the interpretation of canine thoracic radiographs, possibly suggesting the need for further cardiological studies, such as an echocardiographic examination.

The vertebral heart score (VHS) is the most widely used system for assessing cardiomegaly on canine thoracic radiographs (19). However, it can be influenced by the canine morphotype considered. For istance, the reference intervals for some canine breeds (e.g., Cavalier King Charles Spaniel or Chihuahua) (20–22) are different from those of the general canine population. Furthermore, body structure, the respiratory phase and the recumbency side used during the radiography all influence VHS (23). In such a scenario, the interpretation of thoracic X-rays, especially when evaluating the cardiac silhouette, can be challenging. For the above reasons, some of the potential advantages of deploying the proposed model are to overcome interobserver variability and to standardise thoracic radiographs evaluation among veterinarians.

Interestingly, our model performed better in identifying stage B1 and C + D dogs than stage B2 dogs. A straightforward comparison with the literature is not possible because the cases were classified using different parameters in previous studies. In fact, Li et al. (14) report a high precision (accuracy of 82.7%) in the identification by CNN of LA enlargement. The study cases were classed only according to the presence or absence of LA enlargement as classified by echocardiography, while ACVIM stage was not considered. In Banzato et al. (12), a very high accuracy (AUC = 0.965) in the detection of cardiomegaly is reported. However, in this latter study, the dogs were classified only considering the size of the cardiac silhouette evaluated from thoracic radiographs.

Even if the overall accuracy of the developed system for the B2 and C + D stages was adequate a significant number of B2 (23.6%) and C + D (6.7%) cases was this misclassified as B1 potentially classifying deseased dogs as healthy. The authors believe that the lower overall precision in the case classification resulting in the present study (71%) is related to different factors. Firstly, the cases were divided into three groups, whereas a binary case classification was used in the previous studies. The intrinsic differences between dogs classed as B1, B2 or C + D are smaller compared to the differences existing between dogs showing/not showing cardiomegaly. In the current study, the sample was more homogeneous because only elderly dogs were included and, since MMVD is age-related, it is also possible that this factor may have played a role in the classification results generated by the CNNs developed by the other authors. Lastly, this was a multicentric study, whereas all the previous studies were carried out in a single centre, and differences in image quality might lower CNN accuracy. On the other hand, CNNs created using data from multiple institutions tend to have a higher generalisation ability (24).

Not surprisingly, the worst classification results were obtained for the B2 stage dogs. This can be explained, among other factors, by the greater variability that exists between animals classified in this stage of the disease and animals classified as B1 or C + D. In fact, they range from having a slightly enlarged cardiac silhouette to having a severe cardiomegaly with signs of cardiac remodelling. This aspect was also confirmed by the visual distribution of the cases in the t-SNE graph (Figures 3, 5); in fact, we can immediately perceive the variability of stage B2 dogs from the wide distribution of the dots representing this group.

Lastly, the developed heart CNN had a high accuracy in detecting stage C + D dogs. This aspect of the results is similar to that of a previous study evaluating to what extent AI software could identify canine pulmonary oedema (15). Furthermore, high precision in classifying alveolar and/or interstitial pattern was also previously documented in dogs and cats (9, 11).

In human medicine, Ueda et al. (7) proposed a model based on DL for the diagnosis of mitral regurgitation based on chest radiographs. In addition, a visualisation technique was used to check and confirm that the features learned by the AI algorithm were specifically related to cardiac morphological changes due to disease severity. Furthermore, they found that the sensitivity of the model rose as the severity of mitral regurgitation increased. A direct comparison between the previous study and our results was not straightforward due to the different system of heart disease classification.

In the present study, radiographs from both institutions involved were included in the database and used for algorithm training and testing. Another approach could have been to use the radiographs from one institution as a training and validation set and the radiographs from the other institution as a test set. We chose to mix the two databases because the overall number of radiographs was limited and the size of the two databases was markedly different. Furthermore, CNN performance is also influenced by disease prevalence in the different databases. One of the main problems is what is known as overfitting; models often show very high accuracy in internal tests but fail to generalise when exposed to external data. One of the strategies that can be adopted to overcome overfitting, at least partially, is to use data acquired from multiple institutions for the training (24). Further studies, possibly involving a higher number of veterianary clinics are required to assess the real generalisation ability of the network.

This study does have some limitations. Its retrospective nature did not allow us to establish a more rigorous collection of data. The relatively small size of the database, compared to human studies on similar topics (7), also acted as a limiting factor. On this aspect, we would like to point out that the database size was comparable to other studies developing similar CNNs in dogs (9, 13, 14) and cats (11). Lastly, many stage C dogs were receiving diuretic therapy when the radiographs were taken. Even if cardiomegaly was evident, it could be associated with a normal pulmonary pattern. This factor may have led to misclassification of dogs in the C + D group. Further studies selecting only dogs with acute or chronic decompensated HF should be considered.

## 5. Conclusion

An AI-based algorithm for the automatic staging of dogs affected by MMVD was proposed based on canine thoracic radiographs. This method showed a high accuracy in identifying dogs belonging to stage B1 or C + D stage and a moderate accuracy in the identification of stage B2 dogs. Potentially, the use of a larger dataset could provide greater result accuracy. The heart CNN could stand as a useful support tool for general practitioners when interpreting canine chest radiographs. Nonetheless, more studies with a larger sample size would provide a better insight into the performance of the heart-CNN.

## Data availability statement

The raw data supporting the conclusions of this article will be made available by the authors, without undue reservation.

## Ethics statement

This study was conducted respecting Italian Legislative Decree No. 26/2014 (transposing EU directive 2010/63/EU). As the data used in this study were part of routine clinical activity, no ethical committee approval was needed. Informed consent regarding the treatment of personal data was obtained from owners.

## Author contributions

TB, AZ, CG, HP, and CV conceived the study, performed echocardiographic and radiographic examinations, and drafted the manuscript. MW developed the CNN and drafted the manuscript. DC performed echocardiographic examinations and drafted the manuscript. RV drafted the manuscript. All authors contributed to the article and approved the submitted version.
